# Prevalence and antimicrobial resistance patterns of *Enterococcus* species isolated from laying hens in Lusaka and Copperbelt provinces of Zambia: a call for AMR surveillance in the poultry sector

**DOI:** 10.1093/jacamr/dlac126

**Published:** 2022-12-21

**Authors:** Steward Mudenda, Scott Kaba Matafwali, Sydney Malama, Musso Munyeme, Kaunda Yamba, Patrick Katemangwe, Godfrey Siluchali, Geoffrey Mainda, Mercy Mukuma, Flavien Nsoni Bumbangi, Robert Mirisho, John Bwalya Muma

**Affiliations:** Department of Pharmacy, School of Health Sciences, University of Zambia, Lusaka, Zambia; Department of Disease Control, School of Veterinary Medicine, University of Zambia, Lusaka, Zambia; Clinical Research Department, Faculty of Infectious and Tropical Diseases, London School of Hygiene & Tropical Medicine, London, UK; Department of Biological Sciences, School of Natural Sciences, University of Zambia, Lusaka, Zambia; Department of Disease Control, School of Veterinary Medicine, University of Zambia, Lusaka, Zambia; Department of Pathology & Microbiology Laboratory, University Teaching Hospitals, Lusaka, Zambia; Department of Disease Control, School of Veterinary Medicine, University of Zambia, Lusaka, Zambia; Department of Disease Control, School of Veterinary Medicine, University of Zambia, Lusaka, Zambia; Department of Anatomy and Physiological Sciences, Institute of Basic and Biomedical Sciences, Levy Mwanawasa Medical University, Lusaka, Zambia; Department of Veterinary Services, Central Veterinary Research Institute, Ministry of Fisheries and Livestock, Lusaka, Zambia; Department of Food Science and Nutrition, School of Agricultural Sciences, University of Zambia, Lusaka, Zambia; Department of Medicine, School of Medicine, Eden University, P.O. Box 37727, Lusaka, Zambia; Department of Public Health, St Francis University College of Health and Allied Sciences, Ifakara, Tanzania; Department of Disease Control, School of Veterinary Medicine, University of Zambia, Lusaka, Zambia

## Abstract

**Background:**

The use of antimicrobials in layer poultry production for improved production, growth promotion, prophylaxis and treatment purposes has contributed to the development of antimicrobial resistance (AMR) in poultry. In Zambia, there is a paucity of information on the prevalence and AMR patterns of *Enterococcus* species isolated from laying hens.

**Objectives:**

This study investigated the prevalence and AMR patterns of enterococci isolated in layer hens in Lusaka and Copperbelt provinces of Zambia.

**Methods:**

A cross-sectional study was conducted from September 2020 to April 2021. Three hundred and sixty-five pooled cloacal swab samples were collected from 77 layer poultry farms. Enterococci identification and confirmation were performed using Analytical Profile Index (API 20 STREP) and 16S rRNA sequencing, respectively. A panel of nine antibiotics was used for antibiotic susceptibility testing and interpreted according to the CLSI 2020 guidelines. Data were analysed using SPSS version 23 and WHONET 2020.

**Results:**

A total of 308 (84.4%) single *Enterococcus* species isolates were obtained and showed resistance to tetracycline (80.5%), erythromycin (53.6%), quinupristin/dalfopristin (53.2%), ampicillin (36.72%), vancomycin (32.8%), linezolid (30.2%), ciprofloxacin (11.0%), nitrofurantoin (6.5%) and chloramphenicol (3.9%). The prevalence of enterococci resistant to at least one antibiotic was 99.4% (*n* = 306), of which 86% (*n* = 265) were MDR.

**Conclusions:**

This study found a high prevalence of antimicrobial-resistant enterococci. The presence of MDR requires urgent intervention and implementation of AMR surveillance strategies and antimicrobial stewardship programmes in layer poultry production in Zambia.

## Introduction

Poultry production is a significant source of income and nutrients globally.^1–3^ However, the demand for poultry products, including eggs and chicken meat, has led to the inappropriate use of antimicrobial agents, contributing to the development of antimicrobial resistance (AMR).^4–6^ AMR is a global health threat that affects both animal and human health, compromising food security and increasing morbidity and mortality.^7–11^ Enterococci are among the microorganisms that are resistant to antimicrobials used in poultry.^12–14^ When enterococci are exposed to antimicrobials, they tend to have a high capacity to resist these drugs by horizontal transfer propagated by genetic elements and point mutations.^15^

An increase in the prevalence of antimicrobial-resistant microorganisms in poultry has been worsened by the irrational use of antimicrobials.^16–19^ This has been facilitated by easier access to poultry antibiotics without prescription.^3,20–22^ The access to antimicrobials from illegal drug vendors has contributed to the development of AMR in poultry.^8,23^ This is because farmers are not provided with expert knowledge regarding the use of antimicrobials and the consequences of their inappropriate use for prophylaxis, growth promotion and improved egg production.^7,24^ Therefore, there is a need to consider the public health implications of possible abuse and misuse of antimicrobials in poultry farming, especially in backyard poultries.^25^

Enterococci are normal commensals in the human and animal gastrointestinal tract and usually cause nosocomial infections.^26^ Some infections caused by enterococci include endocarditis, intra-abdominal infections, urinary tract infections, diarrhoea, septicaemia and bacteraemia.^27–29^ Moreover, *Enterococcus* spp. have a high capacity to become resistant to antimicrobials used in humans and food-producing animals.^6,30,31^ The continuous misuse of antimicrobials in poultry production has led to antimicrobial-resistant microorganisms such as *Enterococcus* spp., *Escherichia coli*, *Staphylococcus aureus*, *Salmonella* spp., *Klebsiella* spp., *Streptococcus* spp. and *Campylobacter* spp.^4,6,32–34^ These antimicrobial-resistant microorganisms can be transmitted to humans through the food chain.^6,13,35^ Thus, they can cause infections in humans that may be difficult and expensive to treat, may prolong hospitalization, and increase morbidity and mortality.^23,36^

Globally, studies have reported the prevalence of antimicrobial-resistant enterococci in poultry.^12,13^ In Poland, a study reported a prevalence of enterococci isolates of 88.1% from broilers and 5.3% from layers.^37^ Isolated enterococci were resistant to sulfamethoxazole/trimethoprim (88%), tylosin (71.4%), enrofloxacin (69.4%), doxycycline (67.3%), lincomycin (56.1%) and vancomycin (0.12%).^37^ In Canada, *Enterococcus* spp. were resistant to tolincomycin (80.3%), tetracycline (65.3%), penicillin (61.1%) and ciprofloxacin (49.6%).^12^ This shows that enterococci have become resistant to the most common antibiotics used in the treatment of infections in poultry and humans.

In Africa, antimicrobial-resistant enterococci have been isolated from poultry. A study in South Africa reported antimicrobial-resistant enterococci at a prevalence of 56%, with 27.95% being MDR, and increased resistance to tetracycline, quinupristin/dalfopristin and chloramphenicol.^38^ In Zambia, antimicrobial-resistant microbes have been isolated in the food chain.^39–43^ A lack of awareness of AMR and associated factors continues to be a challenge leading to farmers misusing and abusing antibiotics.^44^ Moreover, there is insufficient information on the prevalence and AMR patterns of enterococci isolated from laying hens on Zambian farms. Therefore, this study investigated the prevalence and AMR patterns of *Enterococcus* spp. isolated from laying hens in the Lusaka and Copperbelt provinces of Zambia.

## Materials and methods

### Study design and site

**Figure 1.**
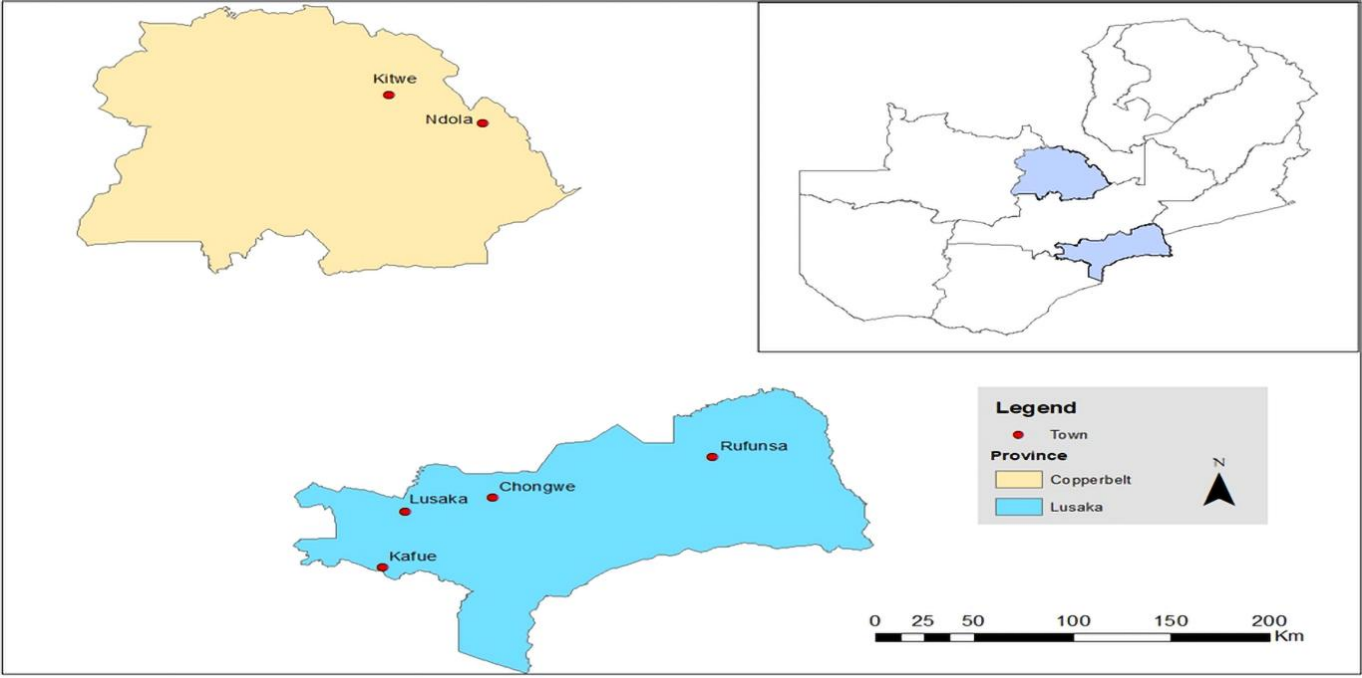
Map of Zambia indicating the sampling sites.

This cross-sectional study was conducted from September 2020 to April 2021 to investigate the AMR patterns of enterococci isolated from layer hens. Cloacal swab samples were collected from layer birds in the Copperbelt and Lusaka provinces of Zambia. Two districts (Kitwe and Ndola) were sampled from the Copperbelt Province, whereas four districts (Chongwe, Kafue, Lusaka and Rufunsa) were sampled from the Lusaka Province. The two provinces were selected because they contribute to the majority of poultry production in Zambia, as reported by the Poultry Association of Zambia (PAZ).^45^ Figure 1 shows the map of Zambia with selected provinces and sampling sites.

### Study population and sampling

This study was conducted on layer poultry farms where owners consented to be part of the study. Multistage sampling was applied in which the districts in Lusaka and Copperbelt provinces were firstly categorized based on their farming practices and activities. A purposive sampling technique was then employed to select six districts in Lusaka Province and two in the Copperbelt Province. Only farms with layer hens at production age were eligible to participate in the study. We excluded sick chickens and those that were being treated with antibiotics at the time of the study.

In each district, layer farmers were identified with the help of Veterinary Assistants, District Veterinary Officers (DVOs) and registers from the PAZ. Layer poultry farms were categorized into three groups based on their bird-rearing capacity: small-scale (≤1000 birds), medium-scale (1001 to 10 000 birds) and large-scale (>10 000 birds). With approximately 96 (*n* = 56 for Lusaka; *n* = 40 for Copperbelt) layer poultry farms in the study areas based on the DVOs and PAZ registers and from a previous study,^44^ the sample size was calculated at a 95% confidence level, 5% desired precision estimate and 50% estimated proportion using Ausvet Epitools (https://epitools.ausvet.com.au/) as was used in a similar study.^32^ Because the identified number of farms was small we conducted a full enumeration, which resulted in an enrolment of 77 layer farms. From each farm, laying chickens were randomly sampled from a poultry house (independent study unit). One cloacal swab sample was collected per 25 m^2^ from each poultry house. Overall, 365 samples were collected. The freshly collected samples were placed and pre-enriched in 10 mL buffered peptone water (BPW) (Oxoid, Basingstoke, UK) and transported to the Public Health Laboratory at the University of Zambia, School of Veterinary Medicine within 8 h of collection for processing and analysis.

### Quality control

Quality control (QC) of media was done at preparation indicating the brand and gross appearance of the powder media used, colour and texture of media, and amount weighed. The amount of distilled water dissolved in and the pH of the broth were measured. After preparation of media QC was done for performance of the media, whereby the physical appearance (e.g. presence of precipitates or wrinkling), sterility and use of known ATCC 29212 strains were determined. The antimicrobial susceptibility testing (AST) internal QC was done using known ATCC strains (i.e. *Enterococcus faecalis* ATCC 29212 and *E. coli* ATCC 25922) according to the CLSI 2020 guidelines.^46^

### Isolation and identification of enterococcal isolates

The pre-enriched samples were incubated at 37°C for 24 h. Selective enrichment was done by adding 1 mL BPW to 9 mL azide dextrose broth (Oxoid, Basingstoke, UK), mixed using a vortex and incubated aerobically at 37°C for 18–24 h. A loop full of broth from azide dextrose broth was then inoculated on Bile Aesculin Azide (BEA) agar (Oxoid, Basingstoke, UK) and incubated aerobically at 37°C for 18–24 h. Typical enterococcal colonies on BEA agar hydrolyse aesculin in the presence of bile and turn the media dark brown or black after incubation. After this, the counts and selection of enterococcal typical colonies, which appear small and translucent with zones between brown-black and black, were done. The identification of enterococcal isolates was done using an Analytical Profile Index (API 20 STREP) (bioMérieux^®^, Inc., Durham, NC, USA) test kit.

### Confirmation of enterococcal isolates

Enterococcal isolates were confirmed using 16S rRNA sequencing as described for *Enterococcus* spp.^47^ The DNA extraction from the identified isolates was done using the heat crude method. Pure colonies of the isolates were suspended in 200 µL nuclease-free water and heated at 95°C for 5 min. The suspension was centrifuged at 6000 *g* for 2 min to extract the DNA. The DNA amplification was done by PCR using *Taq* polymerase and the Tuf F (Forward) primers (TAGTGACAAACCATTCATGATG) and Tuf R (Reverse) primers (AACTTCGTCACCAACCGGAAC) (Merck, Germany) in a thermo-cycler. A total of 40 cycles of amplification were done. The amplified DNA was run on agarose gel to confirm amplification. The gel was immersed in 1% Tris-acetate EDTA buffer containing ethidium bromide dye (0.5 mg/mL) for 30 min. Thereafter, the gel images of the amplified *Enterococcus* spp. were generated using the trans-illuminator, which helped to view the band results for the confirmed *Enterococcus* spp.

### Antimicrobial susceptibility testing

AST was performed using the Kirby–Bauer disk diffusion method on Mueller–Hinton agar (Oxoid, Basingstoke, UK)^48–50^ and as reported by the CLSI 2020 guidelines.^46^ The antibiotic discs (Oxoid, Basingstoke, UK) tested included nitrofurantoin 300 µg (nitrofuran), quinupristin/dalfopristin 15 µg (streptogramin), ciprofloxacin 5 µg (quinolone), chloramphenicol 30 µg (amphenicol), tetracycline 30 µg (tetracycline), linezolid 30 µg (oxazolidinone), ampicillin 10 µg (penicillin), erythromycin 15 µg (macrolide) and vancomycin 30 µg (glycopeptide).

A sterile swab was used to pick pure colonies from the confirmed *Enterococcus* spp. on nutrient agar plates and emulsified in 2 mL normal saline. To achieve the recommended 0.5 McFarland standard, the turbidity of the inoculated normal saline was compared with the standardized 0.5 Remel^™^ McFarland turbidity (Lenexa, KS, USA). A sterile swab was then used to inoculate the bacterial suspensions on the Mueller–Hinton agar plates. The inoculated Mueller–Hinton agar plates were incubated at 37°C for 18–24 h. After incubation, the zones of inhibition were measured using a digital vernier calliper, and interpretations were done according to the CLSI 2020 guidelines as Resistant (R), Intermediate (I) and susceptible (S).^46^

### Data analysis

The collected data were entered in Microsoft Excel^®^ 2016 and then analysed using SPSS version 23 and WHONET 2020. The zones of inhibition were interpreted using the CLSI 2020 guidelines as Resistant (R), Intermediate (I) and Susceptible (S).

### Ethical approval

Ethical approval was granted by the ERES Converge Ethics Committee (ref. no. 2019-Dec-004). We also obtained permission from the Zambia National Health Research Authority and the Lusaka and Copperbelt Provincial and District Veterinary Offices. The farmers also consented to our request to collect cloacal swab samples from their farms.

## Results

Overall, 77 layer poultry farms from 6 districts in the 2 provinces were included in the study. A total of 365 cloacal swab samples were collected from laying hens. Of the total cloacal swab samples, 308 tested positive for enterococcal isolates, translating to a positivity rate of 88.4% (Table 1).

**Table 1.** Distribution of samples (*n* = 365) collected from laying hens (farms *n* = 77)

Province	District	Number of farms sampled, *n* (%; 95% CI)	Number of samples collected	Positive isolates	Positivity rates (%)
Lusaka	Change	17 (22.1; 14.0–33.0)	103	86	83.5
	Kafue	20 (26.0; 17.2–37.1)	81	62	76.5
	Lusaka	5 (6.49; 2.67–14.9)	34	32	94.1
	Rufunsa	3 (3.90; 1.23–11.7)	7	7	100
Copperbelt	Kitwe	22 (28.6; 19.4–39.9)	94	78	83.0
	Ndola	10 (13.0; 7.03–22.7)	46	43	93.5
	**Total**	**77**	**365**	**308**	

*Enterococcus* isolates were highly resistant to tetracycline (80.5%), erythromycin (53.6%) and quinupristin/dalfopristin (53.2%) but susceptible to nitrofurantoin (77.6%) and chloramphenicol (71.1%) as shown in Table 2.

**Table 2.** Antimicrobial resistance patterns of *Enterococcus* spp. (*n* = 308)

Antibiotic	*n* (%) R	*n* (%) I	*n* (%) S	% R 95% CI
Ampicillin	113 (36.7)	—	195 (63.3)	31.3–42.4
Chloramphenicol	12 (3.9)	77 (25)	219 (71.1)	2.1–6.9
Ciprofloxacin	34 (11.0)	126 (40.9)	148 (48.1)	7.9–15.2
Erythromycin	165 (53.6)	107 (34.7)	36 (11.7)	47.8–59.2
Linezolid	93 (30.2)	51 (16.6)	164 (53.2)	25.2–35.7
Nitrofurantoin	20 (6.5)	49 (15.9)	239 (77.6)	4.1–10.0
Quinupristin/dalfopristin	164 (53.2)	68 (22.1)	76 (24.7)	47.5–58.9
Tetracycline	248 (80.5)	22 (7.1)	38 (12.3)	75.6–84.7
Vancomycin	101 (32.8)	71 (23.1)	136 (44.2)	27.6–38.4

I, intermediate; R, resistant; S, susceptible.

### MDR, XDR and pandrug-resistant (PDR) isolates

Overall, 2/308 (0.6%) of the isolates were susceptible to all antibiotics (no AMR) whereas 306/308 (99.4%) were resistant to at least one antibiotic. Overall, 265/308 (86.0%; 95% CI: 81.7–89.5) isolates were resistant to three or more antibiotics from different classes, with 75/308(24.4%; 95% CI: 19.9–29.5) and 9/308 (2.92%; 95% CI: 1.52–5.54) isolates being possible XDR and PDR, respectively.

Nearly all the farms, 75/77 (97.4%; 95% CI: 89.9–99.4) had MDR isolates, including all the farms (45/45) from Lusaka Province and 30/32 (93.8%; 95% CI: 77.4–98.5) from the Copperbelt Province.

The most common MDR patterns found were observed with erythromycin, quinupristin/dalfopristin, and tetracycline. The least MDR patterns were observed with erythromycin, linezolid, and nitrofurantoin (Table 3).

**Table 3.** Selected common and less common MDR patterns of *Enterococcus* species

Antimicrobial combination	Number of isolates	Number of antimicrobial classes
AMP, Q/D, TET	1	3
ERY, LZD, NIT	1	3
ERY, Q/D, TET	29	3
AMP, ERY, Q/D, TET	11	4
ERY, LZD, Q/D, TET	9	4
ERY, NIT, Q/D, TET	5	4
CIP, ERY, LZD, Q/D	13	4
AMP, CIP, ERY, Q/D, TET	7	5
AMP, ERY/LZD, Q/D, TET	5	6
CIP, ERY, LZD, NIT, Q/D, TET	6	6
AMP, CIP, ERY, LZD, Q/D, TET	11	6
CHL, CIP, ERY, LZD, Q/D, TET	19	6
CIP, ERY, Q/D, TET	18	4
AMP, CIP, ERY, LZD, NIT, Q/D, TET	2	7
CHL, CIP, ERY, LZD, NIT, Q/D, TET	12	7
AMP, CHL, CIP, ERY, LZD, Q/D, TET	7	7
AMP, CHL, CIP, ERY, LZD, NIT, Q/D, TET	9	8

AMP, ampicillin; CHL, chloramphenicol; CIP, ciprofloxacin; ERY, erythromycin; LZD, Linezolid; NIT, nitrofurantoin; Q/D, Quinupristin/dalfopristin; TET, Tetracycline.

## Discussion

This study investigated the prevalence and AMR patterns of enterococci isolated from layer chickens in Lusaka and Copperbelt provinces of Zambia. This study revealed a prevalence of 84.4% (*n* = 308) of enterococci isolated from laying hens. Nearly all the isolates (99.4%, *n* = 306) were resistant to at least one antibiotic, of which 86% (*n* = 265) were MDR, isolated from almost all (97.4%, *n* = 75) of the farms investigated. The enterococci were highly resistant to tetracycline, erythromycin and quinupristin/dalfopristin. Low resistance was reported with nitrofurantoin and chloramphenicol.

The prevalence of enterococci isolated in this study is higher than that reported in laying hens (5.3%) in a similar study done in Poland.^37^ However, a higher isolation rate of enterococci (96%) was reported in Denmark,^51^ and 96% in Germany.^52^ The differences and inconsistencies in the isolation rate between our study and comparative studies could have been due to experience and technical factors during the collection of cloacal swabs and laboratory analysis. Technical factors such as isolation methods have been reported to affect the isolation rate of enterococci.^53^ Consequently, the high rate of isolated enterococci poses a public health problem, especially if the isolates are resistant to antimicrobials.

The current study found that the highest resistance of enterococci was observed with tetracycline, similar to other study findings.^13,31,38,54–56^ The high resistance of enterococci to tetracycline reported in our study and similar studies could be due to the overuse and inappropriate use of tetracyclines in poultry production and other livestock activities.^37,40^ A recent study in Zambia found that tetracyclines were highly accessed from community pharmacies and used in poultry,^57^ a potential risk for the development of AMR. The high resistance of enterococci to tetracyclines can also be attributed to their ability to acquire foreign genetic material.^13^ In contrast, lower resistance to tetracycline has been reported in Bangladesh.^58^ This low resistance pattern could be due to the lower use of tetracyclines for prophylaxis and improving poultry production in Bangladesh. This is evidenced by previous studies in which highly used antimicrobials in poultry were amoxicillin and ciprofloxacin.^18,59^

Our study also observed that enterococci were highly resistant to erythromycin. Our findings corroborate reports from similar studies in which enterococci were highly resistant to erythromycin.^13,31,52,56,58,60^ The high resistance of enterococci to erythromycin could be due to the broad spectrum and high usage of this drug in poultry,^54^ causing these microbes to easily acquire resistance to antibiotics.^13^ Erythromycin is commonly used in humans, and thus the resistance of enterococci isolated in laying hens to this drug poses a public health problem in humans. Erythromycin is widely used to treat respiratory tract infections in chickens^4^ and humans.^61^ In Zambia, there is a lack of information on the consumption of erythromycin in poultry. However, evidence has shown that this drug is highly accessed from pharmacies without prescriptions.^57^

Our study found that enterococci were highly resistant to quinupristin/dalfopristin. This is similar to findings from other countries; resistance of enterococci to quinupristin/dalfopristin was reported in Italy,^62^ South Africa^38^ and China.^26^ This is of great concern in public health because this drug is used to treat infections that do not respond to other drugs.^26^ Quinupristin/dalfopristin is recommended as a drug of last resort because it is effective against many antimicrobial-resistant pathogens such as *S. aureus* but has significant adverse effects and drug interactions, and is expensive.^63^ Our findings and those reported in other studies confirm that enterococci have developed intrinsic resistance to quinupristin/dalfopristin.^26,64^ Therefore, the use of these antimicrobials must be avoided in laboratory-confirmed *E. faecalis*.

Our study further found a low resistance to chloramphenicol, nitrofurantoin and ciprofloxacin. The low resistance of enterococci to chloramphenicol was also reported in South Africa.^38^ Additionally, lower resistance to these drugs is a positive finding because they are also commonly used in humans to treat urinary tract infections.^65,66^ In contrast, a study in Canada reported a higher resistance to ciprofloxacin, which could have been due to the overuse of fluoroquinolones in the poultry sector.^12^ The inappropriate use of fluoroquinolones in Poland was also reported to contribute to enterococcal resistance to these drugs.^37^

The current study also reported resistance to ampicillin, similar to reports in other studies, although with varying degrees.^38,56^ In Zambia, access to penicillin antibiotics from pharmacies without prescriptions could be a contributing factor to AMR.^57^ The resistance of enterococci to ampicillin is facilitated by point mutations or the transfer of genetic material from one species to another.^28^ This could be the reason why all enterococcal isolates were found to be resistant to ampicillin in Bangladesh.^58^ Our study noted a public health issue because many enterococci were linezolid resistant. This was also reported in China,^60^ Colombia^67^ and South Korea,^54^ in which some enterococci isolated in poultry were resistant to linezolid. This is of concern because linezolid is the drug of last resort for treating enterococcal infections.^68^ However, our findings are contrary to those in other studies in which all enterococcal isolates were susceptible to linezolid.^38,47,56^ In practice, linezolid is not licensed for poultry use. Thus the resistance of enterococcal isolates to this drug calls for a One Health approach in addressing AMR because there are higher chances of resistance transfer from humans to animals and vice versa.^69^

This study further observed a high resistance of enterococci to vancomycin. The existence of vancomycin-resistant *Enterococcus* has been reported in other studies.^14,38,47,56,58^ The influence of overuse of antimicrobials in poultry has contributed to the AMR of enterococci to vancomycin and other antimicrobials.^47^ Documented evidence has shown that enterococci can become resistant to vancomycin by point mutations or by horizontal genetic transfer.^28^ The resistance of enterococci to vancomycin indicates a public health problem that requires the rational use of antibiotics in poultry.

Our study also found a high rate of MDR enterococci, accounting for 86% of all isolates tested for resistance. Alongside this, the evidence of isolation of possible XDR and possible PDR enterococci is of public health concern. The isolation of MDR enterococci has been reported in similar studies.^4,37,47,58,70^ In South Africa, a slightly lower prevalence of MDR enterococci isolated from poultry was reported.^38^ The current findings and comparative studies of a high rate of MDR enterococci can be attributed to the inappropriate use of antimicrobials in poultry.^58,71^ This public concern requires urgent attention because MDR lessens treatment options.^58,72^ This high resistance of enterococci is a public health concern because the resistant pathogens may be transmitted to humans, especially in farms where biosecurity measures are not implemented.^73,74^

This study provided insight into the isolation rate and phenotypic resistance of enterococci isolated from laying hens in Zambia. However, the study was conducted only in two provinces of Zambia, which may affect the generalization of the findings.

### Conclusions

This study found a high prevalence of antimicrobial-resistant enterococci isolated from laying hens in Lusaka and Copperbelt provinces of Zambia. The isolation of MDR enterococci is of public health concern. Therefore, there is a need to regulate the use of antimicrobials in layer poultry production in Zambia and strengthen antimicrobial stewardship and surveillance programmes in this sector.
