# Correction to: Prevalence and antimicrobial resistance patterns of *Enterococcus* species isolated from laying hens in Lusaka and Copperbelt provinces of Zambia: a call for AMR surveillance in the poultry sector

**DOI:** 10.1093/jacamr/dlad027

**Published:** 2023-03-03

This is a correction to: Steward Mudenda, Scott Kaba Matafwali, Sydney Malama, Musso Munyeme, Kaunda Yamba, Patrick Katemangwe, Godfrey Siluchali, Geoffrey Mainda, Mercy Mukuma, Flavien Nsoni Bumbangi, Robert Mirisho, John Bwalya Muma, Prevalence and antimicrobial resistance patterns of *Enterococcus* species isolated from laying hens in Lusaka and Copperbelt provinces of Zambia: a call for AMR surveillance in the poultry sector, JAC-Antimicrobial Resistance, Volume 4, Issue 6, December 2022, dlac126, https://doi.org/10.1093/jacamr/dlac126

The originally published version of this manuscript contained several errors.

In Results, the percentage for the total of single *Enterococcus* species isolates was incorrectly given as 83%. The correct percentage is 84.4%.

In Table 1, the final line of the Positivity rates (%) column was given as 88.4. This was incorrect and has now been removed.

In the Discussion section, the following sentence was incorrect:

This study revealed a prevalence of 88.4% (n = 308) of enterococci isolated from laying hens.

The sentence has been corrected as follows:

This study revealed a prevalence of 84.4% (n = 308) of enterococci isolated from laying hens.

Also in the Discussion section, the following text required an update:

The prevalence of enterococci isolated in this study is higher than that reported in broiler chickens (88.1%) and laying hens (5.3%) in a similar study done in Poland. Similarly, a lower isolation rate in Bangladesh of 62.5% was reported.

This text has been revised as follows:

The prevalence of enterococci isolated in this study is higher than that reported in laying hens (5.3%) in a similar study done in Poland.

